# WOMAC score and arthritis diagnosis predict decreased agricultural productivity

**DOI:** 10.1186/s12891-021-04041-x

**Published:** 2021-02-13

**Authors:** Eliza J. Webber, Tan Tran, Ronald June, Emily Healy, Tara M. Andrews, Roubie Younkin, Justin MacDonald, Erik S. Adams

**Affiliations:** 1 Center for American Indian and Rural Health Equity, Montana State University, Bozeman, MT USA; 2 Department of Mathematical Sciences, Statistical Consulting and Research Services, Montana State University, Bozeman, MT USA; 3 Department of Mechanical and Industrial Engineering, Montana State University, P.O. Box 173485, Bozeman, MT 59717-3485 USA; 4 Montana Department of Labor and Industry, Montana Occupational Health & Safety Surveillance Program, Helena, MT USA; 5 Montana State University Extension – Custer County, Miles City, MT USA; 6 Montana State University Extension – Valley County, Glasgow, MT USA; 7 University of Washington School of Medicine, Seattle, WA USA

**Keywords:** Arthritis, Osteoarthritis, Ranching, Farming, Agriculture, Disability, WOMAC

## Abstract

**Background:**

Arthritis and joint pain are highly prevalent in agricultural (ag) workers. Many ag operations are sustained by a small number of workers, and the disability of even one worker thus contributes to economic hardship. This study investigated associations between joint health in Montana ag workers and economic well-being and work capacity.

**Methods:**

This observational mixed-methods study utilized quantitative survey data and qualitative focus group data. 299 ranchers and farmers in 9 Montana counties completed either an online or paper survey that included participant demographics, joint symptoms, history of arthritis and arthritis type, financial status, work capacity, and the need to rely on others to complete one’s work. The Western Ontario and McMaster Universities arthritis index (WOMAC) survey was completed by those with hip or knee pain. Data were entered into REDCap v8.9.2 for analysis with SAS 9.4, using logistic and linear regression models to detect associations between covariables and to calculate odds ratios and confidence intervals. Focus groups were held with ranchers in two Montana counties, discussing similar topics, and the themes expressed were identified.

**Results:**

87.6% of survey respondents reported joint pain, 47.8% a diagnosis of arthritis, and 22.4% osteoarthritis (OA). A 10-point increase in WOMAC was significantly associated with lower work capacity (OR 2.00; 95% CI [1.58, 2.55], *p* < 0.01), worse financial condition (OR 1.23; 95% CI [1.01,1.48], *p* = 0.04), and increased reliance on others (OR 1.82; 95% CI [1.32, 2.55], *p* < 0.01). An arthritis diagnosis was associated with worsening work capacity (OR 4.66; 95% CI [2.71, 8.01], *p* < 0.01) and increased odds of relying on others (OR 3.23; 95% CI [1.56, 6.66], *p* < 0.01). A diagnosis of OA was significantly associated with decreased work capacity (OR 3.47; 95% CI [1.97, 6.11], *p* < 0.01). Unadjusted for age and BMI, we found a significant association between years spent working in agriculture and joint health, which became non-significant after adjusting for age and BMI. Focus group themes included decreased productivity with increased joint symptoms and a tendency for ranchers to avoid interaction with the health care system.

**Conclusion:**

Poor joint health is associated with economic risk on Montana ranches and farms.

**Supplementary Information:**

The online version contains supplementary material available at 10.1186/s12891-021-04041-x.

## Background

Arthritis is a leading cause of work limitations [1–3] that affected over 54 million individuals in the United States between 2013 and 2015 [4]. Approximately half of those with arthritis experience disability [4]. Farmers and ranchers have been shown to have higher rates of osteoarthritis than the general population [5–7]. Agriculture is of particular interest in this regard, because of our reliance on their products and the impact of disability on production [3, 8, 9]. Agricultural operations are often small, with family farms having only 1–2 workers and often no hired help, [10] so the physical disability of only one person may have a significant effect. This study intends to examine the impact of work limitations on agricultural production.

The etiology behind higher osteoarthritis rates in agricultural workers is unclear. Agriculture is a physically-demanding occupation, and some studies have shown an association between high physical workload and osteoarthritis [11–14]. In a study of hip and knee arthroplasty by occupation, using sedentary occupations as a control group, male farmers had an odds ratio (OR) of 5.1 for knee replacement and 3.6 for hip replacement, higher than four other physically-demanding occupations [15]. Thelin and Holmberg found that farmers had an OR of 3.0 for hip osteoarthritis and 2.1 for osteoarthritis at any joint, compared to urban counterparts [16]. A large-scale Swedish study showed that male farmers had the highest relative risk (RR) among high exposure occupations of hospitalization for hip osteoarthritis, at 3.78, compared to low exposure occupations [14]. Given this strong association between agricultural work and OA, we sought to explore the impact that joint health may have on agricultural productivity.

The first aim of this study was to determine associations between years spent working in agriculture and joint health. The hypothesis being tested is that there would be a correlation between years in agriculture and worsened joint health. The study’s second aim was to investigate the relationship between joint health and the economic well-being of a ranch or farm, by examining the impact of declining joint health on the ability of a rancher or farmer (“ag producer”) to work at their pre-disability level. The hypothesis being tested for our second objective is that we would see a correlation between worsening joint symptoms and worsening work capacity and economic health of the ranch or farm. Our aims were investigated using a survey of ag producers that consisted of demographics and joint health questions designed by the study’s authors and also the standard Western Ontario and McMaster Universities arthritis index (WOMAC) survey. We also conducted focus groups with Montana ranchers to obtain their viewpoints regarding the effect of joint pain and arthritis on their ability to work, interactions with the health care system, and the economics of agriculture. In our mixed-methods approach, we are asking whether there are impactful joint health issues in the agricultural community that are complicated or informed by the social framework of the community.

## Methods

### Surveys

Surveys were sent to farmers and ranchers in the state of Montana between March and July, 2018. Our survey (Additional file 1**Appendix 1**) consisted of 42 questions, and survey responses were confidential. Toward maximizing sample size and ag producer participation, both paper and online surveys were utilized for this study. Online surveys were accessed by participants by sending them an emailed invitation to participate, including a link to the survey. Ag producers in all 9 participating counties received email invitations between 3/19/18 and 4/16/18 to complete the survey online, using email lists kept by Montana State University (MSU) Extension agents. These lists consisted of ag producers in their county with whom the Extension agents had previous email contact. Extension agents also had postal mailing lists of ag producers in their counties, which were compiled over many years through multiple opportunities for repeated contact with ag producers. The study size was determined by the number of ranchers and farmers willing to participate, and our intent was to maximize this number.

Study data were collected and managed using REDCap (Research Electronic Data Capture) v8.9.2, hosted at the Institute of Translational Health Sciences, University of Washington [17, 18]. REDCap is a secure, web-based software platform designed to support data capture for research studies, providing 1) an intuitive interface for validated data capture; 2) audit trails for tracking data manipulation and export procedures; 3) automated export procedures for seamless data downloads to common statistical packages; and 4) procedures for data integration and interoperability with external sources. Individual REDCap data “arms” were created for each participating county, in order to separate data by county. All surveys, whether input online by the participant or entered by the study’s researchers off a paper survey completed by the participant, were kept in the appropriate arm for their county of origin.

A number of factors led to a decision to utilize paper surveys, in addition to online surveys. After the email invitations were sent, the Extension office in Custer County noted that some of the ag producers did not use email, so more producers would be reached by using the postal service. Paper surveys were therefore mailed to all ag producers on the mailing list in Custer County on 4/15/18. After Extension agents in Granite and Liberty Counties were notified of a poor online survey response, these agents stated that their ag producers tended to respond better to mail, so paper surveys were mailed to producers in those counties on 6/8/19 and 7/27/18, respectively. Paper surveys were also completed by ag producers at agricultural events in Valley and Richland Counties in April, 2018, as this was an easily-accessible cohort, and online access was not available at these events. Invitations to complete paper surveys were accompanied by a request to not do so if the recipient had already completed an online survey. Prior to mailing to ag producers, paper surveys were labeled with the appropriate county, and after completion they were mailed by the participant in a pre-paid envelope to the study’s principal investigator (PI). Paper surveys completed at agricultural events were collected by the Extension agent attending the event and mailed to the PI. The PI and one of the co-authors then manually created a REDCap survey for each paper survey and copied the paper data into the electronic format.

All surveys, including the WOMAC, were completed individually by respondents. Considering the size of the state of Montana, and that many of the survey participants lived in remote areas of the state, sometimes as much as 30 miles from the nearest paved road, it was not possible to have researchers present to guide respondents through the survey. The WOMAC was completed by all respondents with hip or knee pain and is a self-administered survey that is widely used and is available in 65 languages, requiring about 12 min to complete. The WOMAC is highly validated, with a Cronbach’s alpha score of 0.86, which indicates good internal consistency [19]. All three subscales (pain, stiffness and function, the greatest proportion of the score being devoted to function) of the WOMAC were included and the total WOMAC score was tabulated in REDCap for each respondent. Regression analysis was performed to detect correlations between WOMAC and self-reported work capacity, as has been validated elsewhere [20].

The inclusion criteria were that the participant be an active rancher or farmer (livestock and/or crops) in one of the study’s participating counties and willing to complete a survey. There were no exclusion criteria, and no statement was included in the survey invitation relating to whether the participant may or may not have joint disease or joint symptoms, nor any other medical condition. The survey asked respondents to list the top two agricultural products from their ranch or farm, and these data were used to analyze for correlations between ag products and joint health indices.

Survey respondents provided information on their demographics, joint pain (“Do you have pain in any joints? yes/no) and arthritis diagnosis status (“Has a doctor diagnosed you with arthritis?” yes/no), work limitations caused by joint pain, and economic impacts. Those with hip or knee pain completed the WOMAC portion on the survey. Surveys were excluded from the analysis if the respondent did not answer key questions about whether they had joint pain. Additional file 1**Appendix 2** shows the participating counties. Respondents were sent a $15 gift card for an online store, in a manner that preserved confidentiality.

### Sample size calculation

Our sample size of 299 provides 0.9 power to detect an association between years working and odds of arthritis diagnosis and a power of 0.8 to detect an association between years working and odds of osteoarthritis diagnosis, respectively, with an effect size of 1.25 for every 10-year increase in years working (*p* = 0.05). Our sample of 181 WOMAC respondents provided a power of > 0.9 to detect an association between years working and mean WOMAC score, with an effect size of 5.0 per 10-year increase in years working.

### IRB approval

This study was approved by the MSU Institutional Review Board, and all participants provided informed consent. We partnered with MSU Extension because of their close ties to the farming and ranching community. MSU Extension has agents and an office in each of 56 Montana counties and 7 Montana reservations that are involved in education and community projects that are both agriculture and non-agriculture related [21].

#### Data analysis

Survey responses were downloaded from REDCap. Data were then cleaned to remove blank survey submissions (missing values for all study variables) and illogical response values. All analyses were conducted using SAS 9.4 (SAS Institute Inc., Cary, NC, United States of America). Descriptive statistics were produced to show overall, unadjusted distribution of study outcomes and population demographics in our sample. Continuous variables were presented as both medians with inter-quartile ranges and means with standard deviations, while categorical variables were presented as numbers and percentages.

#### Clinical outcome variables

In our first aim, we assessed the following joint health outcomes: joint pain (yes/no), arthritis diagnosis (yes/no), osteoarthritis (OA) diagnosis (yes/no) and WOMAC score (continuous). Respondents reporting a diagnosis of arthritis specified the type of arthritis in the next question, which included “osteoarthritis”, “rheumatoid arthritis”, “gout”, “psoriatic”, “lupus”, “Lyme arthritis”, “other”, and “not sure”. The primary predictor was years working. Logistic regression modeled the unadjusted and adjusted effect of years working on joint pain, arthritis diagnosis, and OA diagnosis, while general linear regression modeled the effect of years working on WOMAC score, among those with hip or knee pain. Age and body mass index (BMI) were included as covariates in the adjusted models, using the equations given in Additional file 1**Appendix 3**.

#### Relationships between clinical outcome variables and economic outcomes

Our second aim was to determine the association between joint health and the following economic risk outcomes: financial well-being of the ranch or farm, workload capacity, and reliance on others to perform one’s work. The financial well being question applied to all respondents and asked them to rate the financial well-being of their ranch or farm, out of five possibilities: “doing extremely well,” “doing fairly well,” “more or less breaking even,” “struggling,” or “doing poorly.”

Questions about workload capacity and reliance on others were answered only by those with joint pain. The primary predictor was WOMAC score, among those with hip or knee pain. Presence of an arthritis diagnosis and an OA diagnosis were secondary predictors. Workload capacity measured the percentage of work farmers/ranchers with joint pain were still able to perform, compared to their previous capacity. Categories included “100% of previous capacity”, “50%-75% of previous capacity”, “25%-50% of previous capacity”, “less than 25% of previous capacity”, and “I cannot perform any physical work at all.” A category of “75-99% of previous capacity” was not included, as a distinction was needed between an ability to perform all work (100%) and a minimal level of disability (76–99%). Categories of < 25 and 0% were collapsed in all study analyses, leaving four quartiles of work capacity (76–100%, 50–75%, 25–50%, and less than 25%).

Workload capacity was treated as an ordinal variable, allowing the category of “100%” to represent “75–100%” as a complete continuous ordinal response. Proportional odds regression [22] modeled the odds of moving from a category of workload capacity to the next category of doing less work. In the following set of cumulative probability models, the cumulative probability of “farmer/ranchers being able to perform at <25% or *more* of their previous work capacity” is not shown because it covers all possible categories and equals 1. The model controlled for age, years working, and BMI, as shown in Additional file 1**Appendix 3**.

#### Sensitivity analyses

Sensitivity analyses were conducted to assess differences in joint health outcomes and impact of joint health on economic risk, by farming/ranching type. Farming/ranching type was determined by the top two agricultural (“ag”) commodities produced on the respondent’s farm/ranch. Options included cattle, sheep, dairy, other livestock, small grains/cereal, pulse crops, hay, and other crops. Dairy, other livestock and other crops were not included in the analyses due to small sample size (*N* < 15). Each commodity was included as a binary predictor in the regression models described in Aim 1 to assess impact on 1) joint pain, 2) arthritis diagnosis 3) osteoarthritis diagnosis and 4) WOMAC score, controlling for age, years working and BMI. Next, Aim 2’s models, assessing impact of WOMAC score, arthritis diagnosis and OA diagnosis on economic risk were re-run, stratified by top farming/ranching commodity.

#### Integration of qualitative data

Qualitative data were obtained from focus groups. These were assembled in Custer and Valley Counties by invitation from the agricultural Extension agents, consisting of 6–7 ranchers per county. Invitations were made to particular ranchers, based on who the Extension agents believed would be willing to share their opinions freely. Meetings were led by asking the questions shown in Additional file 1**Appendix 4**, and encouraging participants to share their own experiences and perceptions of the agricultural community. Audio recordings from the meetings were transcribed, and two researchers conducted a formal content analysis of the transcripts. Audio recordings and corresponding transcripts were reviewed in their entirety by two of the researchers. Preliminary themes and representative quotes were independently identified using an inductive “bottom up” approach in which codes were developed from the data. Preliminary themes were then compared and further refined until consensus was reached between the two researchers. These qualitative results were then aligned with conclusions from quantitative survey data, as a means to provide explanation for the observed correlations. The intent of the use of our qualitative data in a mixed methods approach is to add meaning and context to the quantitative findings from the survey data [23, 24].

## Results

### Survey results

We received 304 surveys, 213 of which were in paper format. Four online surveys were returned with no data, and one paper survey with a non-sensical answer to years spent working. These five surveys were deleted. Duplication of a paper survey was detected with one respondent, who returned five surveys showing identical data, in identical handwriting; these survey data were entered only once. This left 299 surveys for analysis, 212 of which were in paper format and 87 online. For those questions not requiring a conditional previous response (e.g., only if you have joint pain …) missing responses occurred at a mean rate of 2.42 (0.81%) per question (SD 1.92) with number of workers being members of the respondent’s family (2.01% missing), weight (2.01% missing), height (1.67% missing), financial wellbeing of the farm or ranch (1.34% missing) and number of employees hired (1.34%) comprising the questions with highest missing response rates. Survey responses by county are shown in Additional file 1**Appendix 2**, with cumulative data by county for a selection of survey questions. Response rates were calculated as number of surveys completed per email address or mailing address invited to participate. In the three counties that received mailed surveys, the mailing list for ag producers contained more producers than the email list and also resulted in a better response rate. In Liberty County, 124 emailed invitations resulted in 6 completed surveys (4.9%), whereas 192 mailed invitations resulted in 95 (49.4%), for a total response rate of 52.6% for Liberty County. The corresponding emailed/mailed response rates for Custer and Granite Counties were 18.9%/34.5 and 28.6%/50.0% per email or mailing address, respectively. Among the counties that received only emailed invitations, response rates ranged from 4.3 to 14.2%. The number of emailed, mailed and in-person (agricultural event) survey invitations, and completed surveys by county are included in Additional file 1**Appendix 2**.

Out of the 299 survey respondents, 87.6% experienced joint pain, 47.8% had been diagnosed with arthritis, and 22.4% had a diagnosis of OA. Among those with hip and knee pain (*n* = 123), median WOMAC score was 23 (IQR: 13, 34). Median respondent age was 62 (IQR: 54, 68), median number of years working on farm/ranch was 40 (IQR: 27, 48), and the median BMI was 27 (IQR: 25, 31). Top produced commodities were cattle (64.6%), small grains/cereal (40.8%) and hay (38.5%). The majority of farmers/ranchers reported doing fairly well (51.8%) or extremely well (11.7%) financially. Among those with joint pain, 45.8% reported a reduction in workload capacity, and 27.4% needed to rely on others to perform previous pre-joint pain duties (Table 1) .

**Table 1:** Descriptive Statistics of Survey Respondents

Variable	Total (***n*** = 299)^a^	
Joint Health Outcomes		
Joint pain	n (%)	262 (87.63%)
Arthritis diagnosis	n (%)	143 (47.83%)
Osteoarthritis diagnosis	n (%)	67 (22.41%)
WOMAC score^b^	Median (IQR)	23 (13, 34)
	Mean (STD)	24.38 (15.24)
Economic Risk Outcomes		
Workload, compared to previous capacity		
100% of previous capacity	n (%)	121 (40.47%)
50–75% of previous capacity	n (%)	99 (33.11%)
25–50% of previous capacity	n (%)	22 (7.36%)
Less than 25% of previous capacity	n (%)	16 (5.35%)
Rely on others to perform previous duties	n (%)	82 (27.42%)
Financial wellbeing of farm/ranch		
Extremely well	n (%)	35 (11.71%)
Fairly well	n (%)	155 (51.84%)
Breaking even	n (%)	92 (30.77%)
Doing poorly	n (%)	13 (4.35%)
Demographic information		
Age	Median (IQR)	62 (54, 68)
	Mean (SD)	59.59 (14.13)
Years working on ranch	Median (IQR)	40 (27, 48)
	Mean (SD)	37.72 (16.35)
Body mass index (BMI)	Median (IQR)	27 (25, 31)
	Mean (SD)	28.15 (4.24)
Top commodities produced on farm/ranch		
Cattle	n (%)	193 (64.55%)
Sheep	n (%)	26 (8.70%)
Other livestock	n (%)	16 (5.35%)
Small grains/cereal	n (%)	122 (40.80%)
Pulse crops	n (%)	48 (16.05%)
Hay	n (%)	115 (38.46%)
Other crop	n (%)	13 (4.35%)

^a^Numbers and percentages may not add to 100% of total due to missing values

^b^Among those experiencing joint pain in hips or knees (n = 123)

**Fig. 1:**
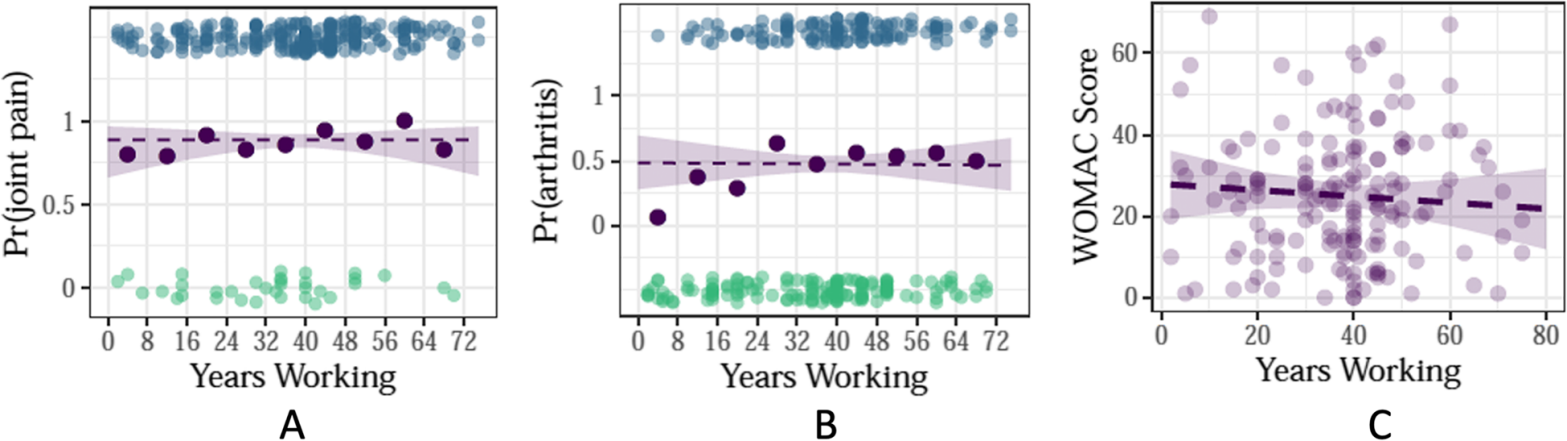
Aim 1 primary outcomes. Adjusted for age and BMI, no associations were detected between years working in ranching or farming and **A**, the probability of reporting joint pain; **B**, the probability of reporting a diagnosis of arthritis; **C** – the WOMAC score. In Figures 1**A** and 1**B**, the observed binary responses are clustered at the upper and lower edges of the plots (lower: no joint pain or no diagnosis of arthritis; upper: positive joint pain or diagnosis of arthritis). In Figure 1**C**, the dashed line is the estimated association between years worked and mean WOMAC score, adjusted for age and BMI, with 95% confidence bands. Data points are added for reference.

After adjusting for age and BMI, no evidence of association was observed between number of years working on a farm/ranch and the following joint health outcomes: presence of joint pain (OR 1.00; 95% CI [0.70, 1.42], *p* = 0.99), arthritis diagnosis (OR 0.99; 95% CI [0.79, 1.24], *p* = 0.93), OA diagnosis (OR 0.83; 95% CI [0.64, 1.07], *p* = 0.28) and WOMAC score (mean difference: -0.38; 95% CI [− 2.41, 1.65], *p* = 0.71) per 10-year increase in years working (Table 2 and Fig. 1a-c).

**Table 2:** Effect of Years Working^a^ on Joint Pain, Arthritis Diagnosis, and WOMAC

Outcomes	Unadjusted			Adjusted^b^		
	Estimate^c^	95% CI	*P*-value	Estimate	95% CI	P-value
**Joint Pain**	1.29	[1.04, 1.60]	0.02	1.00	[0.70, 1.42]	0.99
**Arthritis Diagnosis**	1.26	[1.08, 1.45]	< 0.01	0.99	[0.79, 1.24]	0.93
**Osteoarthritis Diagnosis**	1.09	[0.92, 1.31]	0.16	0.83	[0.64, 1.07]	0.28
**WOMAC**	0.47	[−0.97, 1.91]	0.52	-0.38	[−2.41, 1.65]	0.71

^a^Per 10 unit increase in years^b^Adjusted models include age and BMI as coefficients

^c^Estimates are presented as Odds Ratios (OR) for joint pain and arthritis diagnosis and geometric means for WOMAC

Table 3 demonstrates the associations between WOMAC score, arthritis diagnosis, and OA diagnosis on economic risk outcomes, overall and stratified by top ag commodity. Overall, every 10-point increase in WOMAC score among those with hip or knee pain was associated with double the odds of reporting a next lower work capacity in the survey (OR 2.00; 95% CI [1.58, 2.55], *p* < 0.01), significantly higher odds of moving to the next worse financial category (OR 1.23, 95% CI [1.01, 1.48], *p* = 0.04), and significantly higher odds of needing to rely on others to complete farm/ranch duties (OR 1.82, 95% CI [1.32, 2.55], *p* < 0.01). The association between higher WOMAC scores and reduced workload capacity remained across all ag commodity types. Associations between WOMAC and other economic risk outcomes were more varied across commodity types. Higher WOMAC scores were significantly associated with needing to rely on others among cattle farmers (*p* < 0.01) small grain/cereal farmers (*p* = 0.02) and hay farmers (*p* = 0.01), while an association between WOMAC score and self-report of decreased financial well-being was only present in pulse crop farmers (*p* = 0.02).

**Table 3:** Adjusted Effect of WOMAC Score and Arthritis Diagnosis on Economic Risk Outcomes^a^, Overall and Stratified by Top Farming/Ranching Commodity

Outcomes Measuring Economic Risk	WOMAC^c^			Arthritis Diagnosis			Osteoarthritis Diagnosis		
	Estimate^b^	95% CI	P-value	Estimate^b^	95% CI	P-value	Estimate^b^	95% CI	P-value
**Overall**									
Lower workload capacity	2.00	[1.58, 2.55]	< 0.01	4.66	[2.71, 8.01]	< 0.01	3.47	[1.97, 6.11]	< 0.01
Lower financial well-being status	1.23	[1.01, 1.48]	0.04	1.02	[0.65, 1.62]	0.92	0.83	[0.48, 1.42]	0.49
Reliance on others	1.82	[1.32, 2.55]	< 0.01	3.23	[1.56, 6.66]	< 0.01	1.07	[0.54, 2.13]	0.85
**Cattle Ranchers**									
Lower workload capacity	1.95	[1.44, 2.64]	< 0.01	6.65	[3.26, 13.54]	< 0.01	3.11	[1.51, 6.40]	< 0.01
Lower financial well-being status	1.13	[0.87, 1.45]	0.38	0.92	[0.52, 1.65]	0.79	0.36	[0.17, 0.78]	0.01
Reliance on others	2.71	[1.52, 4.81]	< 0.01	3.11	[1.27, 7.65]	0.01	0.65	[0.26, 1.60]	0.35
**Sheep Ranchers**^d^									
Lower workload capacity	6.04	[1.37, 26.73]	0.02	5.53	[0.42, 72.38]	0.19	11.80	[0.93, 150.56]	0.06
Lower financial well-being status	2.28	[0.94, 5.55]	0.07	0.71	[0.10, 4.78]	0.72	1.44	[0.25, 8.34]	0.69
Reliance on others	1.82	[0.49, 6.67]	0.37	1.23	[0.07, 21.06]	0.89	1.45	[0.13, 16.44]	0.77
**Small Grain/Cereal Farmers**									
Lower workload capacity	1.54	[1.12, 2.10]	0.01	3.40	[1.44, 8.01]	0.01	2.68	[1.14, 6.28]	0.02
Lower financial well-being status	1.24	[0.95, 1.64]	0.12	1.15	[0.55, 2.43]	0.71	1.04	[0.45, 2.39]	0.92
Reliance on others	1.74	[1.12, 2.74]	0.02	6.22	[1.68, 23.09]	0.01	1.93	[0.62, 5.99]	0.25
**Pulse Crop Farmers**^d^									
Lower workload capacity	3.64	[1.45, 9.17]	0.01	3.29	[0.72, 14.99]	0.12	1.82	[0.44, 7.57]	0.41
Lower financial well-being status	2.04	[1.15, 3.64]	0.02	1.54	[0.40, 5.97]	0.53	2.77	[0.60, 12.79]	0.19
Reliance on others	2.48	[0.89. 7.01]	0.08	7.04	[0.83, 59.81]	0.07	5.97	[0.51, 69.83]	0.15
**Hay Farmers**									
Lower workload capacity	2.64	[1.68, 4.15]	< 0.01	4.87	[1.99, 11.89]	< 0.01	3.91	[1.50, 10.16]	0.01
Lower financial well-being status	1.33	[0.92, 1.93]	0.12	0.96	[0.46, 2.01]	0.91	0.85	[0.33, 2.15]	0.73
Reliance on others	2.74	[1.26, 5.94]	0.01	2.27	[0.70, 7.32]	0.17	0.43	[0.11, 1.63]	0.21

^a^Results from regression models, adjusted by age, years working, and BMI^b^Estimates are expressed as Cumulative Odds Ratios for “lower workload capacity “and “lower financial wellbeing status “, and as Odds Ratios for “reliance on others”

^c^Estimates show multiplicative change in odds per 10-unit increase in WOMAC score

^d^Because of the low number of respondents producing these commodities, stratified analyses are limited in power and data for these commodities should be considered dubious

Overall, ranchers and farmers with diagnosed arthritis had a nearly 5 times higher odds of reduced work capacity (OR 4.66; 95% CI [2.71,8.71], *p* < 0.01), and significantly higher odds of needing to rely on others to complete farm/ranch duties (OR 3.23; 95% CI [1.56,6.66], *p* < 0.01), compared to those without an arthritis diagnosis. Among those with diagnosed OA, an association with diminished work capacity remained (OR 3.47, 95% CI [1.97, 6.11, *p* < 0.01], compared to those without, but no significant difference was observed in needing to rely on others. Overall, no difference in financial well-being status was observed between those with and without an arthritis diagnosis (OR 1.02, 95% CI [0.65, 1.62], *p* = 0.92] or OA diagnosis (OR 0.83, 95% CI [0.48, 1.42], *p* = 0.49). When stratified by ag commodity, associations between arthritis and OA diagnosis and diminished work capacity remained in cattle ranchers (*p* < 0.01), small grain/cereal farmers (*p* = 0.01 for arthritis, *p* = 0.02 for OA) and hay farmers (p < 0.01 for arthritis and *p* = 0.01 for OA). Having an arthritis diagnosis was positively associated with needing to rely on others among cattle ranchers (*p* = 0.01) and small grain/cereal farmers (*p* = 0.01). An OA diagnosis was associated with higher odds of doing well financially in cattle ranchers (*p* = 0.01). Table 3 demonstrates that, across all commodities, there is 95% confidence in the odds of those with hip and knee pain experiencing an increasingly higher odds of moving to the next lower quartile of work capacity with every 10-point increase in WOMAC. Excluding sheep ranchers and pulse crop farmers, similar associations with reduction in work capacity were observed when comparing diagnosis status of arthritis and OA, respectively, by commodity type. No other significant associations were observed between joint health and economic risk, stratified by farming/ranching commodity type.

### Focus group results

Two researchers independently identified the following themes and sub-themes: lack of access to healthcare (sub-themes: time pressures, cost, skepticism, cultural value of toughness), unavailability of help (sub-themes: younger generation leaving ranching, hired help difficult to find), ranching is a lifelong occupation, and physical demands of ranch work (sub themes: demands of cattle ranching, physical toll on body, technological advances in equipment). Quotes extracted from the focus group transcriptions are included in Additional file 1**Appendix 5** to help illustrate the themes and sub-themes, and Additional file 1**Appendix 6** illustrates the themes and sub-themes diagrammatically.

Lack of access to health care in ranchers contained both external factors (distance, cost) and internal factors (a culture of toughness in ranching, time pressures of ranching, and skepticism about the value of visiting a physician). Ranchers, especially female ranchers, expressed that physicians do not take their pain seriously and did not appreciate the physical demands of ranching. Because of its long recovery period and the impact on ranch work ( “… that’s going to put me out of business for 4 to 6 months”), joint replacement is often delayed until disability is severe, resulting in ranchers working in pain for many years. Treatment recommendations are often not followed because of the demands of ranching ( “… the sling only stayed on one day”, “… you don’t follow up with physical therapy afterwards, because you’re so darn busy.”), and follow-up visits may not occur because of time constraints, distance and cost. Ranchers also expressed that they are working into older age and may not have the help of their grown children, who are more apt lately to find occupations outside of agriculture ( “… you used to have a pile of kids (to help).”), and that hired help is difficult to find in rural areas.

## Discussion

### Joint health and economic health in agriculture

A second stated aim of this study was to assess the effects of joint pain and arthritis on the economics of farming and ranching. For those with hip or knee pain, we found higher WOMAC scores to be negatively associated with a farm or ranch’s financial well-being, with every 10-point increase in WOMAC score increasing the odds of self-reporting the next worse financial category by a factor of 1.23. When a ranch or farm is operated by only a small number of persons, such as in our study (mean = 2.8) and that of McMillan et al. (73% of farms had 1–2 workers), [8, 10] the physical disability of one person is apt to cause decreased productivity, which may lead to economic hardship. The study by McMillan et al. found a high incidence of work-interrupting musculoskeletal pain in their cohort of 2595 Saskatchewan farmers, with 27.9% experiencing pain that interrupted work, but they did not investigate whether there was a correlation between disability and productivity. Our study found this correlation. For every 10-point increase in WOMAC in ranchers and farmers, the odds of moving to the next worse quartile of work capacity increased by a factor of 2.0, and odds of needing to rely on others to perform one’s work increased by a factor of 1.8. We also found that those diagnosed with arthritis or OA had statistically-significant odds ratios of 4.66 and 3.47, respectively, of moving to the next worse quartile of work capacity. Respondents reporting a diagnosis of arthritis but not specifying OA had a statistically-significant OR of 3.23 for needing to rely on others to do their work. A linkage between musculoskeletal disorders and diminished work capacity was echoed in our qualitative focus group results.

Our focus group results pointed to a culture of toughness that allowed ranchers to work through pain when injured, and our quantitative survey data certainly show diminished productivity in the presence of symptoms. We did not hear of any rancher missing work entirely. A Swedish study by Holmberg et al. [25] of 1013 full-time farmers with 769 non-farmer controls, matched for age, sex and geographic area, showed significantly greater musculoskeletal symptoms in the farmers, but no greater utilization of health care for those problems, as well as less sick leave. Work capacity was not assessed in their survey. The phenomenon of farmers going to work despite pain matches our sub-theme of a culture of toughness obtained from our focus group data.

In our study, for all types of agricultural commodities, a diagnosis of arthritis or OA was similarly associated with lowered work capacity, and an arthritis diagnosis was associated with increased reliance on others. These findings are consistent with prior studies and provide further evidence of an association between arthritis and reduced work capacity in ag producers [3, 8]. With regard to ranchers as a subset of ag producers, our focus group results illuminate the importance of this association. Coordinating these quantitative results with our qualitative data, ag producers may find themselves in the difficult position of being unable to do the work themselves and simultaneously losing the younger generation to non-farm work while being unable to hire additional help. One rancher in a focus group observed that the workload gets cut down to what they can do themselves; this workload would necessarily diminish in the presence of musculoskeletal disability. The situation is then complicated by our focus group theme of an avoidance of medical care (sub-themes of time pressures, cost, skepticism, and cultural value of toughness). Our mixed-methods approach shows a linkage between quantifiable demonstration of the effects of musculoskeletal disability on production, and the social forces that complicate these issues. This is concerning, with regard to the economic well-being of ag producers and our reliance on their commodities.

Our study found no association between diagnosed arthritis or OA and the self-reported economic condition of the ranch or farm, but these self reports may have been unreliable. A literature review on the determinants of social desirability bias in surveys found income to be a particularly sensitive survey topic due to the social context of the question [26]. Two recent studies comparing differences in self-reported and actual income found strong evidence for social desirability bias in self-reported income, resulting in inflated values [27, 28]. We were told by MSU Extension agents that farmers and ranchers were unlikely to provide accurate responses to questions about financial health. Therefore, we relied more on the surrogate indicators of reduced work capacity and the need to rely on others to perform work.

Of those diagnosed with arthritis in our study, 35.3% were unsure of the type, and many likely had OA, it being the most common arthritis type in the United States, with 12.1% of adults meeting diagnostic criteria [29]. When calculating associations between OA diagnosis and economic hardship outcomes, those with diagnosed OA were compared to those without, the latter category including no arthritis diagnosis, those with other types of arthritis and those with arthritis but unsure of the type. This misclassification of cases likely weakened or nullified the true associations between OA and outcome variables in our study. Despite this, our study observed a significant association between having OA diagnosis and reduced work capacity. Complete inability to work was rare in our survey respondents, a phenomenon that was echoed in our focus groups and also shown in previous studies [30, 31]. This is likely attributable to the culture of toughness and qualities of perseverance demonstrated in our focus group themes.

### Arthritis prevalence

One of our study aims was to determine the prevalence of doctor-diagnosed arthritis and OA among Montana ag workers. However, our focus group results with ranchers highlighted barriers to accessing medical care in this population, which ranged from geographic and economic difficulties to outright avoidance (“What are they (doctors) going to do?”, “You just let it heal crooked.”). As such, cases may be vastly under-reported due to these healthcare barriers contributing to under-diagnosis within the ag community. More research is needed to further explore the presence of these barriers and associated consequences within the broader ag community.

Arthritis prevalence in our study was higher than was seen in the Montana Behavioral Risk Factor Surveillance Survey (MT-BRFSS), which samples respondents randomly by phone [32]. Unadjusted for age, our study showed a higher arthritis prevalence in agricultural workers (47.0% of respondents) than the MT-BRFSS (20.9% of respondents). After age adjustment, using the age distribution of the Montana population, these values were 32.5 and 18.1%, respectively. Selection bias in our study may have been encouraged by the inclusion of a cover letter sent with each survey or survey invitation that represented this project as a first step in an effort to improve arthritis care in the agricultural community, which may have attracted more respondents who have joint issues. The MT-BRFSS [32] showed ag workers to have a slightly lower prevalence of arthritis than the general population (age-adjusted ag 18.1%, non-ag 19.9%), which disagrees with literature studies [14–16]. As with our survey, the MT-BRFSS inquired whether respondents had been diagnosed with arthritis by a health professional. These findings do not account for those who have arthritis but have not been diagnosed, and considering the common avoidance of medical care expressed by ranchers in our study, may under-estimate the true prevalence of arthritis in this population.

The mean age of ranchers and farmers in our survey was higher than that obtained in MT-BRFSS (59.3 years, S.D. 14.3 and 49.9 years, S.D. 16.7, respectively) [32]. The majority of our surveys were conducted in paper format in Custer, Granite and Liberty counties. For Custer and Granite, two surveys were included in each mailing, and the mean ages for survey respondents in those counties were 65.0 and 62.7, respectively. To test whether we were only obtaining surveys completed by the oldest workers (e.g., the parents) on the ranch or farm, when perhaps their grown children may still work the farm but live separately, we included five surveys in each paper survey mailing for Liberty County. These surveys were marked in a fashion that maintained confidentiality but allowed grouping of surveys from each individual farm or ranch. Using the reported number of workers on a farm/ranch, when all workers from a farm/ranch submitted a survey, the mean age was 51.7, close to that of the MT-BRFSS cohort. In comparison when not all workers completed surveys on a given farm/ranch in Liberty County, the mean age was 60.8. This difference was statistically significant (*p* < 0.01). The inclusion of only two surveys per mailing in Custer and Granite Counties likely selected for an older working population, explaining most of the age difference when compared to MT-BRFSS.

Addressing Aim 1 of our study, we found an association between cumulative years working in agriculture and joint pain and a diagnosis of arthritis (OR 1.29 and 1.26, respectively, for every 10 years spent working in agriculture). After adjusting for age and BMI**,** this association disappeared (Table 2). This was unexpected, as time spent working in a high physical workload occupation has been shown in other studies to be an independent predictor of hip or knee osteoarthritis [14, 33] or total hip or knee arthroplasty [15]. In our focus groups, participants expressed that agriculture is a lifelong occupation (“Born and raised on a farm and ranch and done that pretty much all my life.”), which would create a close association between age and years spent in agricultural work. Our survey results show a mean respondent age of 59.3 and a mean of 37.6 years spent working in agriculture, indicating that most of our respondents had spent their adult years on a ranch or farm. Our inability to detect an association between years working and joint health may be explained by the highly colinear relationship between age and years working in our sample, resulting in type 2 error.

Although the WOMAC portion of our survey is well-tested, the remainder was constructed de novo for this study. We could find no established survey that combined the elements we were seeking, especially regarding economic health. We feel that our survey and its results add insight into the plight of ranchers and farmers and the connection between musculoskeletal disability and ranch or farm productivity.

We believe that our results are not generalizable to the overall population but are specific to farmers and ranchers. However, considering our reliance on their commodities, the demonstration of a correlation between joint health and work capacity highlights the need for effective musculoskeletal treatment strategies in the ag population.

## Conclusions

For Montana ranchers and farmers, this mixed methods study demonstrated a statistically-significant association, but not causality, between having a diagnosis of arthritis or OA or a worsening WOMAC score and factors that may indicate economic risk to an agricultural operation. Our survey respondents demonstrated a reduced work capacity and a need to rely on others to complete agricultural tasks in the presence of worsening knee or hip symptoms (WOMAC score) or a diagnosis of arthritis or OA. Ranchers in our focus groups described a culture of toughness and working through pain, along with avoidance of medical care. Coupled with the younger generation seeking employment outside of agriculture, this leaves an older population struggling to maintain ranch and farm productivity in the presence of musculoskeletal disability. More effective treatment strategies for this population are therefore needed, tailored to the needs of the agricultural community and the nature of their daily work.

### Limitations

This study was vulnerable to selection bias, as ag producers with joint symptoms or joint disease may be more likely to participate, due to interest in addressing their disorder. We did not consider this to be a modifiable source of bias, as our Extension agents believed it was important to explain our intent to the ag community, as an issue of trust. Our survey invitation therefore explained our desire to study joint problems in the ag community and to eventually find novel ways of addressing those problems. The likelihood of selection bias is supported by a higher arthritis prevalence in our study than in the MT-BRFSS, which was a random telephone sampling. As discussed above, self reports of financial condition are also prone to inaccuracy. We resorted to using work capacity, a need to rely on others to complete one’s work, or a need to hire others as surrogates for economic productivity. While the WOMAC survey is well-validated and widely used, the remainder of our survey has not been validated, and true correlations between WOMAC and our other survey data may therefore differ from those determined by our analyses. An additional limitation, although common and accepted in the literature, [6, 32] is self-reporting of diagnoses by study subjects. In addition to the possibility of arthritis under-diagnosis among a population that tends to avoid medical care, subjects may not always accurately remember their diagnoses.

## Supplementary Information

**Additional file 1.**

## Data Availability

The datasets supporting the conclusions of this article are available at the Dryad repository, 10.5061/dryad.0p2ngf1wm

## Abbreviations

OA: Osteoarthritis
MSU: Montana State University
OR: Odds ratio
REDCap: Research Electronic Data Capture
WOMAC: Western Ontario and McMaster University osteoarthritis index
MT-BRFSS: Montana Behavioral Risk Factor Surveillance Survey
ag: agriculture or agricultural
