# Supplementary material for: WOMAC score and arthritis diagnosis predict decreased agricultural productivity
Source: BMC Musculoskelet Disord. 2021 Feb 13;22:181. doi: 10.1186/s12891-021-04041-x (PMC7882068; doi:10.1186/s12891-021-04041-x)
Supplement: Supplementary file 1 — Additional file 1. [file 12891_2021_4041_MOESM1_ESM.docx]

Appendix 1. Survey questions

Thank you for participating in this survey. This survey is part of a collaborative effort between MSU Extension and the INBRE research program at Montana State University. Our hope is to direct more attention to effective arthritis care in Montana’s ranchers and farmers. All responses are confidential.

Participation is voluntary. You can stop at any time. You can choose to not answer any questions you do not wish to answer. By completing the survey you are granting us permission to use your responses in our research.

Please leave blank any questions that are not applicable to your situation.

# In the last 10 years, what have been your top two commodities:

# Cattle Sheep Dairy Other livestock:

# Small grains/cereals Pulse crops Hay

# Other crop:

# How many persons perform physical work on your ranch or farm, on any given day?

# How many of these persons are members of your family (relatives)?

# Do you own your ranch or farm? Yes No

# Please indicate the percentage of your work hours spent on your ranch or farm vs. away from the ranch or farm (the two values should add up to 100):

# Percentage of work hours on ranch/farm:

# Percentage of work hours away from ranch/farm:

# How many years have you been ranching or farming?

# Please indicate your age:

# Male Female

# Please indicate your weight:

# Please indicate your height:

# Do you have health insurance? Yes No

# Please indicate your marital status:

# Single Married Divorced

# Please indicate your education level:

# Did not graduate high school High School Some college

# Associates Degree Bachelor’s degree Graduate degree

# Other:

# Please rate the financial well-being or your ranch or farm: 1. Doing extremely well 2. Doing fairly well 3. More or less breaking even 4. Struggling 5. Doing poorly

# Do you have pain in any joints? Yes No

# During how many days of the month do you have joint pain?

# 1 to 6 7-12 13-18 19-24 25 to all days

# Has a doctor diagnosed you with arthritis? Yes No

# If you have arthritis, which type? osteoarthritis rheumatoid arthritis gout

# psoriatic (psoriasis-related) lupus Lyme arthritis other:

# not sure

# Did a doctor obtain x-rays of your painful joint(s)? yes no

# In which joint(s) have you been diagnosed with arthritis? (check all that apply) none knee hip shoulder neck (cervical spine)

# low back (lumbar spine) wrist or hand ankle or foot

# elbow other joint:

# In which joint(s) do you have pain but have not been diagnosed with arthritis? (check all that apply) not applicable knee hip shoulder neck (cervical spine)

# low back (lumbar spine) wrist or hand ankle or foot

# elbow other joint:

# Have you had a joint replacement? (check all that apply) no joint replacement knee hip shoulder wrist or hand

# ankle or foot other:

# Have you had spine surgery? Yes No

# If you had a joint replacement, do you still have another arthritic joint that causes symptoms? I never had a joint replacement Yes (check all applicable joints) No knee hip shoulder elbow wrist or hand ankle or foot

# neck (cervical spine) low back (lumbar spine) other joint:

# If you had a joint replacement, did it improve your ability to perform physical work? I never had any problems performing physical work before my joint replacement. major improvement minor improvement no improvement

# If you have joint pain now, to what extent does it limit your ability to perform physical work? 1: I can still perform all work 2: I can work at between 50 and 75% of my previous capacity 3: I can work at between 25 and 50% of my previous capacity 4: I can work at less than 25% of my previous capacity 5: I cannot perform any physical work at all

# If your joint pain limits your ability to work, what types of activity are affected? (check all that apply) Work on horseback Driving a 4-wheeler Operating heavy machinery Lifting and carrying Driving Walking Climbing Feeding and care of livestock Other:

# If your joint pain limits your ability to work, did you have to begin relying on others to perform your previous duties? (leave blank if no limitations) Yes No

# If your joint pain limits your ability to work, did you need to hire additional employees (leave blank if no limitations) Yes No

# If you hired additional help, did this cause you financial hardship? (leave blank if did not hire additional employees) Yes No

# Has the financial well-being of your ranch or farm been affected by your work limitations? (leave blank if no limitations) Yes No

# On a scale of 1 to 5, if work limitations have affected your ranching or farming operation financially, to what extent? (leave blank if no work limitations) 1: No financial impact

# 2: Minimal impact – it has changed a few of our decisions 3: Moderate impact – financially challenging

# 4: Substantial impact – our financial survival is questionable 5: Severe impact – our farm or ranch cannot survive because I cannot work

# Does your joint pain and its impact on your work create concern that your ranch or farm may not be able to remain in the family?

# Yes No Ranch or farm not owned by my family

# Do you think your joint pain affects your mood? Yes No

# Describe how your joint pain affects a typical day of working on your ranch or farm:

# Whom do you see for arthritis treatment? Medical doctor (M.D. or D.O.) Nurse practitioner or physician’s assistant Chiropractor Naturopath Nutritionist Other:

# Do you take pain medication for your joint pain? Yes No

# If you take pain medication, does it improve your ability to work? Yes No

# How many days a week do you take pain medication for joint pain? 0 1-2 3-4 5-6 7

# Is at least one of your pain medications an opioid (such as hydrocodone, codeine, tramadol, oxycodone)? Yes No

# If you take opioid pain medications for your joint pain, how many days a week do you take this medication? 0 1-2 3-4 5-6 7

# On a scale of 1 to 5, how satisfied are you with previous treatment for your joint symptoms? Have not had treatment

# 1: not at all 2: minimally 3: somewhat 4: moderately 5: very satisfied

# ______________________________________________________________________________

# Only if you have joint pain in your hips or knees, please fill out the following standardized Western Ontario and McMaster University survey. Please check only one box for each question:

# Think about the pain you felt in your hip or knee in the past 48 hours. How much pain did you have?

|  | none | mild | moderate | severe | extreme |
| --- | --- | --- | --- | --- | --- |
| Walking on a flat surface |  |  |  |  |  |
| Going up and down stairs |  |  |  |  |  |
| At night while in bed, pain disturbs your sleep |  |  |  |  |  |
| Sitting or lying down |  |  |  |  |  |
| Standing upright |  |  |  |  |  |

# Think about the stiffness (not pain) you have in your hip or knee in the past 48 hours. Stiffness is a sensation of decreased ease in moving your joint

|  | none | mild | moderate | severe | extreme |
| --- | --- | --- | --- | --- | --- |
| How severe is your stiffness after first awakening in the morning? |  |  |  |  |  |
| How severe is your stiffness after sitting, lying down, or resting during the day? |  |  |  |  |  |

# Think about the difficulty you have had in doing the following daily physical activities due to your hip or knee during the last 48 hours. By this we mean your ability to move around and look after yourself. What degree of difficulty do you have?

|  | none | mild | moderate | severe | extreme |
| --- | --- | --- | --- | --- | --- |
| Descending stairs |  |  |  |  |  |
| Ascending stairs |  |  |  |  |  |
| Rising from sitting |  |  |  |  |  |
| Standing |  |  |  |  |  |
|  | none | mild | moderate | severe | extreme |
| Bending to the floor |  |  |  |  |  |
| Walking on flat surfaces |  |  |  |  |  |
| Getting in or out of a car, or on or off a bus |  |  |  |  |  |
| Going shopping |  |  |  |  |  |
| Putting on your socks or stockings |  |  |  |  |  |
| Rising from the bed |  |  |  |  |  |
| Taking off your socks or stockings |  |  |  |  |  |
| Lying in bed |  |  |  |  |  |
| Getting in or out of the bath |  |  |  |  |  |
| Sitting |  |  |  |  |  |
| Getting on or off the toilet |  |  |  |  |  |
| Performance heavy domestic duties |  |  |  |  |  |
| Performance light domestic duties |  |  |  |  |  |

| Appendix 2. Cumulative data by county for a selection of survey questions | | | | | | | | |  | |  | |  | |  | |  | |  | |
| --- | --- | --- | --- | --- | --- | --- | --- | --- | --- | --- | --- | --- | --- | --- | --- | --- | --- | --- | --- | --- |
| **Montana county** | | **Custer n=82**  **75/197^2^** | | **Gallatin n=4**  **93/0^2^** | | **Granite n=36**  **28/56^2^** | | **Judith Basin n=16**  **146/0^2^** | | **Liberty  n=86**  **124/192^2^** | | **Park n=30**  **280/0^2^** | | **Powder River n=6**  **210^3^** | | **Richland n=9**  **49/0/X^4^** | | **Valley n=30**  **30/0/45^4^** | | **Total n=299** |
| Top two ag commodities |  | Cattle/ Hay | | Cattle/ Hay | | Cattle/ Hay | | Cattle/ Hay | | Small grains, cereals/ Pulse crops | | Cattle/ Hay | | Cattle/ Sheep | | Cattle/ Small grains, cereals | | Cattle/ Small grains, cereals | | Cattle/ Small grains, cereals |
| Number of workers per farm/ranch | mean (SD) | 2.4 (1.2) | | 2.3 (1.9) | | 3.2 (1.3) | | 2.8 (1.2) | | 3.2 (1.1) | | 2.3 (1.4) | | 3.5 (1.4) | | 2.2 (0.8) | | 2.6 (2.5) | | 2.8 (1.4) |
|  | median (IQR) | 2.0  (2.0, 3.0) | | 1.5  (1, 3.5) | | 3.0  (2.0, 4.0) | | 2.5  (2.0, 4.0) | | 3.0  (2.0, 4.0) | | 2.0  (1.0, 2.0) | | 3.5  (2.0, 5.0) | | 2.0  (2.0, 3.0) | | 2.0  (2.0, 3.0) | | 2.0  (2.0, 4.0) |
| Number of workers that are family | mean (SD) | 2.0 (1.2) | | 1.8 (1.0) | | 2.5 (1.3) | | 2.4 (1.4) | | 2.7 (1.1) | | 1.6 (1.2) | | 2.7 (1.2) | | 2.0 (0.9) | | 1.8 (1.2) | | 2.2 (1.2) |
|  | median (IQR) | 2.0  (1.0, 3.0) | | 1.5  (1.0, 2.5) | | 3.0  (2.0, 3.0) | | 2.0  (1.5, 3.5) | | 3.0  (2.0, 4.0) | | 1.0  (1.0, 2.0) | | 2.5  (2.0, 4.0) | | 2.0  (1.0, 3.0) | | 2.0  (1.0, 2.0) | | 2.0  (1.0, 3.0) |
| Age | mean (SD) | 64.1 (10.9) | | 60.3 (12.7) | | 62.7 (11.9) | | 49.5 (16.6) | | 58.3 (15.0) | | 58.4 (13.1) | | 53.2 (14.9) | | 61.3 (16.4) | | 54.4 (16.6) | | 59.6 (14.1) |
|  | median (IQR) | 65.0  (58.0, 71.0) | | 62.5  (51.5, 69.0) | | 63.0  (57.0, 69.0) | | 45.0  (40.0, 65.0) | | 60.0  (53.0, 66.0) | | 60.5  (57.0, 66.0) | | 53.5  (38, 63) | | 66.0  (64.0, 69.0) | | 59.0  (47.0, 66.0) | | 62.0  (54.0, 68.0) |
| Male gender | % | 86.4 | | 50.0 | | 61.1 | | 75.0 | | 58.1 | | 80.0 | | 0 | | 44.4 | | 70.0 | | 68.8 |
| Body mass index | mean (SD) | 29.2 (4.0) | | 27.7 (4.7) | | 28.0 (4.2) | | 28.4 (4.1) | | 28.3 (4.3) | | 26.8 (4.6) | | 28.6 (7.5) | | 26.4 (3.1) | | 26.8 (3.7) | | 28.1 (4.2) |
|  | median (IQR) | 28.0  (27.0, 32.0) | | 26.0  (24.0, 33.0) | | 27.5  (25.0, 32.0) | | 27.0  (25.0, 32.0) | | 28.0  (26.0, 30.0) | | 26.5  (24.0, 29.0) | | 26.0  (26.0, 31.0) | | 26.0  (25.0, 28.0) | | 26.5  (25.0, 29.0) | | 27.0  (25.0, 31.0) |
| Joint pain | % | 92.7 | | 75.0 | | 91.7 | | 75.0 | | 87.2 | | 86.7 | | 100 | | 88.9 | | 80.0 | | 87.9 |
| Arthritis diagnosis | % | 46.3 | | 50.0 | | 44.4 | | 43.8 | | 51.2 | | 53.3 | | 66.7 | | 55.6 | | 36.7 | | 47.8 |
| Osteoarthritis diagnosis | % | 15.85 | | 50.0 | | 25.0 | | 25.0 | | 25.58 | | 20.0 | | 50.0 | | 33.33 | | 16.67 | | 22.41 |
| Joint pain, limited workload | % | 53.3 | | 33.3 | | 50.0 | | 28.6 | | 64.0 | | 46.1 | | 75.0 | | 66.7 | | 39.1 | | 53.1 |
| *Rely on others* | % | 61.9 | | 0 | | 52.9 | | 11.1 | | 54.6 | | 39.1 | | 60.0 | | 57.1 | | 50 | | 50.9 |
| *Hired additional help* | % | 21.4 | | 0 | | 23.5 | | 10.0 | | 31.0 | | 20.8 | | 20.0 | | 28.6 | | 7.7 | | 22.2 |
| *Hired help, financial hardship* | % | 50.0 | | 0 | | 50.0 | | 0 | | 57.1 | | 35.7 | | 66.7 | | 33.3 | | 50.0 | | 41.8 |
| *Work limitations, financial hardship* | % | 56.8 | | 0 | | 41.2 | | 0 | | 53.5 | | 27.3 | | 16.7 | | 42.9 | | 54.6 | | 43.2 |
| *Concern of losing family farm^1^* | % | 18.6 | | 0 | | 15.6 | | 7.1 | | 14.7 | | 21.4 | | 0 | | 22.2 | | 18.2 | | 16.3 |
| Joint pain affects mood | % | 72.2 | | 0 | | 81.3 | | 60.0 | | 80.8 | | 65.5 | | 66.7 | | 66.7 | | 82.6 | | 74.8 |
| Taking joint pain medication/opioids | % | 63.9/8.0 | | 66.7/0 | | 73.5/0 | | 42.9/0 | | 76.4/8.9 | | 55.2/7.4 | | 83.3/16.7 | | 77.8/14.3 | | 62.5/7.1 | | 67.3/6.9 |
| 1. Among those who own their ranch or farm. 88.2% of respondents owned their ranch or farm. | | | | | | | | | | | | | | | | | | | | |
| 1. Emailed/mailed survey invitations, by county. | | | | | | | | | | | | | | | | | | | | |
| 1. Mailed postcard invitations with a link to the online survey. No mailed paper surveys. | | | | | | | | | | | | | | | | | | | | |
| 1. Emailed/mailed/ag event survey invitations, by county. In Richland County, it is unknown how many persons were at the ag event; in Valley County, there were 45. | | | | | | | | | | | | | | | | | | | | |
|  | | |  | |  | |  | |  | |  | |  | |  | |  | |  | |

Appendix 3. Statistics method

The following equations were used in Aim 1 to model joint health outcomes (joint pain, arthritis diagnosis, OA diagnosis – all yes/no, and WOMAC score, a continuous variable) as a function of age, years working and BMI.

*logit*(*πjointpain*) = *β*0 + *β*1(*Years Working*) + *β*2(*BMI*) + *β*3(*Age*)

*logit*(*πarthritis*) = *β*0 + *β*1(*Years Working*) + *β*2(*BMI*) + *β*3(*Age*)

*logit*(*πosteoarthritis*) = *β*0 + *β*1(*Years Working*) + *β*2(*BMI*) + *β*3(*Age*)

mean(*WOMAC*) = *β*0 + *β*1(*Years Working*) + *β*2(*BMI*) + *β*3(*Age*)

For Aim 2, which investigated associations between joint health (WOMAC, arthritis diagnosis, OA diagnosis) and economic risk factors (financial well-being of the ranch/farm, workload capacity, and reliance on others to perform one’s work), the proportional odds regression model used the following equations.

*Y1* = *Pr*(farmer can do 75% or *more* of their work)

$$\log\left( \frac{Y_{1}}{1-Y_{1}} \right)=\theta_{1}-B_{1}\left( WOMAC \right)-B_{2}\left( BMI \right)-B_{3}\left( years working \right)-B_{4}\left( age \right)$$

*Y2* = *Pr*(farmer can do 50% or *more* of their work)

$$\log\left( \frac{Y_{2}}{1-Y_{2}} \right)=\theta_{2}-B_{1}\left( WOMAC \right)-B_{2}\left( BMI \right)-B_{3}\left( years working \right)-B_{4}\left( age \right)$$

*Y3* = *Pr*(farmer can do 25% or *more* of their work)

$$\log\left( \frac{Y_{3}}{1-Y_{3}} \right)=\theta_{3}-B_{1}\left( WOMAC \right)-B_{2}\left( BMI \right)-B_{3}\left( years working \right)-B_{4}\left( age \right)$$

Where $\theta_{j}$ for j=1-3 is the intercept term for each respective sub-model.

An arthritis or an OA diagnosis were substituted for WOMAC in the above equations to determine the effect of those variables, and the model controlled for BMI, years working and age.

Proportional odds was also used to model the cumulative probability of a farmer/rancher moving to a lower level of financial well-being. Cumulative probabilities were summed as follows:

*Y1* = *Pr*(farmer doing extremely well financially)

$$\log\left( \frac{Y_{1}}{1-Y_{1}} \right)=\theta_{1}-B_{1}\left( WOMAC \right)-B_{2}\left( BMI \right)-B_{3}\left( years working \right)-B_{4}\left( age \right)$$

*Y2* = *Pr*(farmer doing fairly well or better financially)

$$\log\left( \frac{Y_{2}}{1-Y_{2}} \right)=\theta_{2}-B_{1}\left( WOMAC \right)-B_{2}\left( BMI \right)-B_{3}\left( years working \right)-B_{4}\left( age \right)$$

*Y3* = *Pr*(farmer breaking even or better financially)

$$\log\left( \frac{Y_{3}}{1-Y_{3}} \right)=\theta_{3}-B_{1}\left( WOMAC \right)-B_{2}\left( BMI \right)-B_{3}\left( years working \right)-B_{4}\left( age \right)$$

Where $\theta_{j}$ for j=1-3 is the intercept term for each respective sub-model. The cumulative probability of a “farmer doing poorly or better financially” covers all four categories so it is ignored in the model.

Reliance on others was a binary indicator of whether respondents with joint pain needed to rely on others to perform their duties. Logistic regression modeled the odds of needing to rely on others to perform previous work duties.

logit(πreliance_on_others) = β0 + β1(WOMAC) + β2(BMI) + β3(Age) + β4(Years Working).

Appendix 4. Focus Group Questions

1. What do you see as the top health concerns within the Montana agricultural community?
2. Do you believe that resources are adequate to address these concerns? If not, how could they be improved?
3. Do health issues harm the economic health of ranches or farms? If so, which health problems, and how do they exert their harm?
4. How serious of a problem is arthritis in the agricultural community?
5. Do joint replacements work well as an option for arthritis, in terms of returning ranchers and farmers to work?
6. MSU has acquired a motor home that has been outfitted as a mobile research vehicle? How would you like this to be used in the agricultural community?
7. We are proposing doing stem cell injections for knee arthritis in the mobile research vehicle. These procedures are commonly performed in outpatient clinics. What are your thoughts regarding this?
8. Working with joint pain – how does your community manage?
9. Aging agricultural community – implications for health, productivity, health issues forcing early retirement

These meetings will also present an opportunity to obtain community ideas regarding INBRE’s new mobile research vehicle, HERB (Health Education and Research Bus)

Additional HERB (Heath Education Research Bus, our mobile research vehicle) questions:

1. Do you see ways in which this vehicle might be useful within your community?
2. Would the vehicle duplicate, detract from, or compete with services or resources already being provided in your community?
3. How might this vehicle from MSU be perceived within your community? Positively? Negatively?
4. What do you think of the proposed uses for the vehicle: service, outreach, research? Are there others we should consider?
5. Are there potential problems, barriers, or issues that we may not have considered?

Appendix 5. Focus group themes and sub-themes

| **Theme** | **Sub-theme** | **Quotes** |
| --- | --- | --- |
| Lack of access to health care | Time pressures | “And a lot of these ranchers, they want to continue to ranch. And when they start talking about a replacement, their shoulder, hip, knee, their first thing is, ‘ah, that’s going to put me out of business for 4 to 6 months. There ain’t no way I’m replacing anything.’”  “If you drive to Billings, I mean, it’s basically a two-day event. Sometimes you just can’t be gone that long.”  “Or if you do (go to the doctor), you don’t follow up with physical therapy afterwards, because you’re so darn busy.” |
|  | Cost | “…$10,000 or $15,000 deductible, and that’s hard for me to come up with.”  “You have to be bleeding pretty bad before you’re going to go…especially since insurance has skyrocketed.”  “You just let it heal crooked.” |
|  | Skepticism | “Yeah, spend $250 to take three more Advil.”  “The doctors don’t really, they don’t take rural people seriously.”  “And then our daughter who’s in PA school says, ‘Well, Dad, why don’t you go to the doctor?’ And I said,… ‘Well, what are they going to do?’” |
|  | Cultural value of toughness | “We don’t go to the doctor.”  “…it was calving season, so the sling only stayed on one day.”  “No whining, no crying.”  “Get ‘er done. Cowboy up.”  “It’s called living through the pain.” |
| Unavailability of workers | Younger generation leaving ranching | “…if we look at the last 20 years, the age of our farming community has really increased…”  “…and so, we have older individuals doing the work that the young guys…used to do…And that’s wearing us out.”  “And you used to have a pile of kids.”  “Our kids, our grandkids, watch what we go through, and…that’s probably why they find better jobs.”  “I’m 54. When I started, yep, nobody was this old.” |
|  | Hired help difficult to find | “…there’s no workers to be hired. You can’t find any.”  “We’ve been looking for 20 years.”  “And some of them you get, you have to go out and supervise them. You might as well be doing it youself.” |
| Ranching is a lifelong occupation |  | “I was raised on a ranch, have been on a ranch all my life, except for when I was in school. Now, I’m working with my husband on the ranch.”  “I’ve packed 5-gallon buckets of grain since I was, you know, 8-9 years old.”  “Delivered my first set of twin lambs at age 6.” |
| Physical demands of ranch work | Demands of cattle ranching | “Last Spring, calving had to be the worst of all, with just continual cold, miserable weather.”  (In response to question of most demanding agricultural activity):  “Calving”  “Calving”  “Calving”  “You start thinking about it, the...what you do with branding, and... ...shipping time ...that's when our injuries tend to happen around…”  “When you’re working around [calves] heads, and up with your knees. I think that’s where my knee problems come from. It took the cartilage right out of my knees, because It’s up and down three-, four-hundred times that day.” |
|  | Physical toll on body | “I’d rather ride a horse for 20 miles than sit in a tractor for an hour.” (discussing constantly turning head to look behind while plowing)  “You're in a hurry, you're short handed, and you're tired…And then all of the sudden, the drill stem is sticking out of your foot or something like that.”  “Agriculture is one of the most dangerous professions. A lot of people get hurt and killed every year. PTO shaft or whatever, and boom, it’s over. Machinery and animals too. You get kicked or run over. It’s dangerous.”  “Last winter, we had to shovel corn out of the back of our pick-up, and so you’re always bent over, then, tossing every damn morning, so that was exhausting. Makes you feel fairly old.” |
|  | Technological advances in equipment | “I know when we were kids, it was, when they were talking about these small bales, and lifting the hay without the equipment, that was almost slave work. Like my Dad – how he could lift three hundred bales…a day.”  “I do know that I don’t…do half the work I used to be doing. I mean with modern machinery…”  “One of the biggest advancements is the equipment…now, everything is hydraulic, handling the hay.” |

Appendix 6. Diagrammatic representation of focus group themes and sub-themes. Themes in bold; sub-themes in smaller boxes.

| 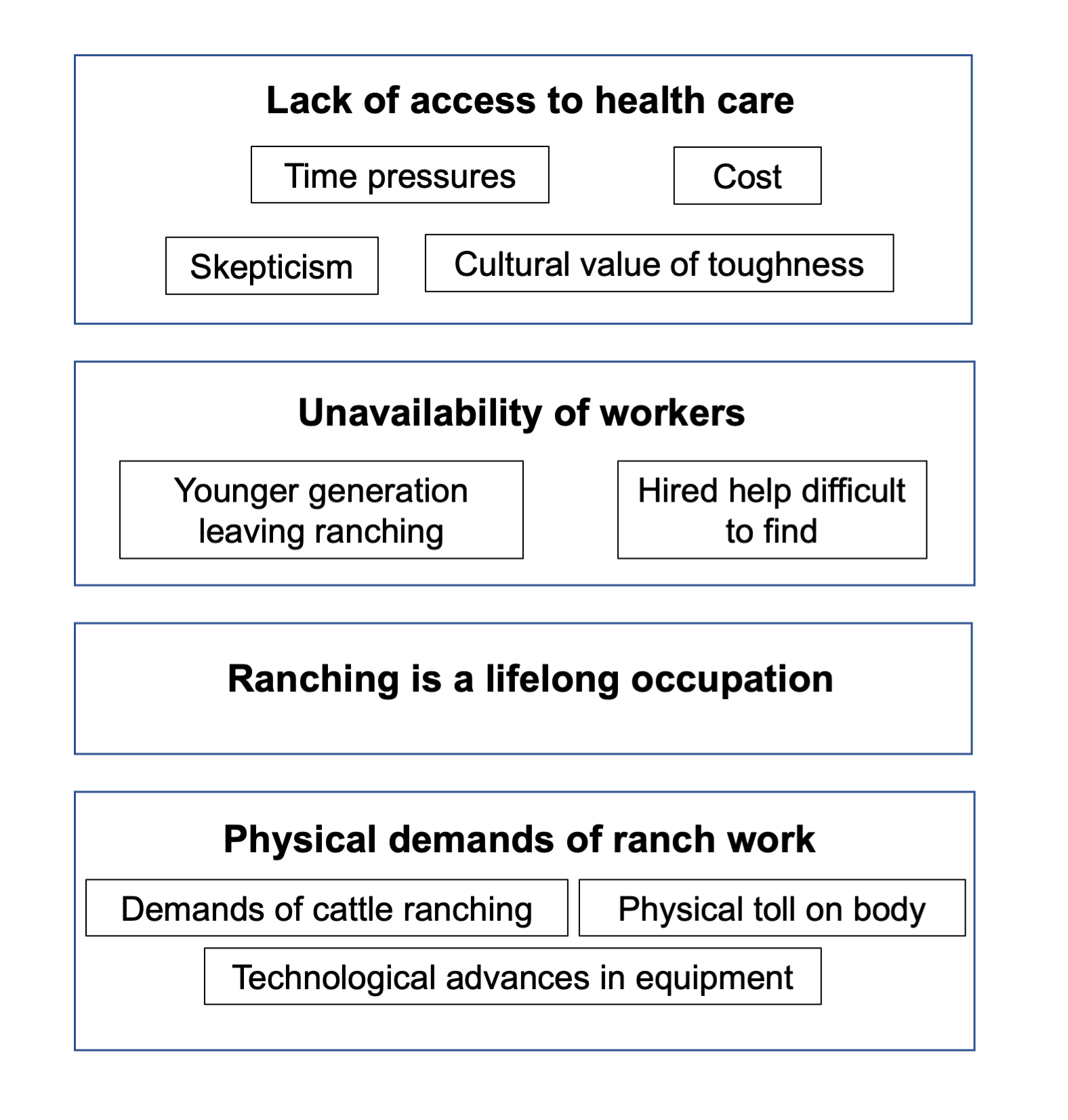 |
| --- |
